# Editorial: Computational methods for neuroimaging: Challenges and future trends

**DOI:** 10.3389/fncom.2023.1181169

**Published:** 2023-03-30

**Authors:** Deepak Gupta, Utku Kose, Victor Hugo C. de Albuquerque

**Affiliations:** ^1^Department of Computer Science & Engineering, Maharaja Agrasen Institute of Technology, New Delhi, India; ^2^Department of Computer Engineering, Suleyman Demrel University, Isparta, Türkiye; ^3^Telecommunication Engineering Graduate Program, Federal Institute of Ceara, João Pessoa, Brazil

**Keywords:** neuroimage, computational method, neuroinformatics, brain mapping, neuro-engineering

Computational methods have been proposed as a useful and effective framework for analyzing brain activity. Enabling a direct communication pathway between the brain and external devices (brain computer/machine interfaces) is necessary for light of the significant difficulties associated with processing brain signals obtained from neuroimaging modalities. While there has been an increasing interest in these questions, the contribution of fuzzy systems has been diverse depending on the area of application. The processing of extremely noisy signals likely affected by non-stationarities, invariants, and poor generalization presents a significant challenge when decoding brain activity. However, advanced computational intelligence methods that handle uncertainty, such as fuzzy sets and systems, represent an excellent tool for overcoming this challenge. Yet, in neuroscience, the concepts of possibility and fuzziness have been used similarly to gauge the degree of coordination between neurons, synapses, and other brain areas. The proposed Research Topic seeks to fill the need for a dedicated platform where experts in the field of computational intelligence may come together to discuss how they might model and communicate the inherent uncertainty in the processing of neuroimaging data. Neuroscience encompasses many subfields, including but not limited to: computational neuroscience; brain-computer/machine interfaces; neuroscience; neuroinformatics; neuroergonomics; computational cognitive neuroscience; affective neuroscience; neurobiology; brain mapping; neuro-engineering; and neurotechnology.

Computational approaches used in neuroimaging are the subject of this Research Topic, which explores recent developments, problems, and future views from various academic disciplines. Therefore, we encourage researchers to contribute to this Research Topic with new, original work that takes advantage of the recent methodology using computational and mathematical techniques in neuroimaging, addresses the challenges of developing dedicated systems for various clinical applications, and proposes novel ideas and directions for future development.

In this Research Topic, experts from various fields discuss current developments, difficulties, and potential directions for applying computational approaches in neuroimaging. Twenty-six articles were submitted to this section, but only five were chosen for review. There were at least two reviewers and two review rounds for each submission. Below, we provide a quick overview of the publications' respective contributions.

In the first paper of this Research Topic, Popova et al. present an opinion about Aphasia. Aphasia is a neurological disorder often caused by lesions in language-related areas of the left hemisphere after a stroke. One of the primary aims of aphasia research is to deconstruct the intricate processes of language reacquisition. Familiarity with these mechanisms is essential for providing reliable prognoses for patients with post-stroke Aphasia. These individuals make up a diverse population with widely varying responses to treatment.

Alsubai et al. proposes a hybrid Convolutional Neural Network-Long Short Term Memory (CNN-LSTM), for classifying and predicting brain tumors using MRI. Authors have used an MRI brain image dataset and the data have first preprocessed, then, the Convolutional Neural Network (CNN) is applied to the preprocessed data to extract the significant features. The proposed CNN-LSTM model predicts the brain tumor with an important classification accuracy of 99.1%.

In this paper by Borkar et al., the authors proposes a model for the estimation of the chronological age of a healthy person from the resting state brain activation (rsfMRI). Authors proposes an age prediction pipeline Ayu which consists of data preprocessing, feature selection, and an attention-based model for deep learning architecture for brain age assessment. The results predict a classification accuracy of 72.619%, a mean absolute error of 6.797, and an r2 of 0.754 as compared to the state-of-the-art approach.

In this research, Oku et al. proposes the bootstrap modularity test to determine whether a pair of brains is co-activated. In this work, the graph hub measures the dyad's synchronization which is critically explained by the relation between the teacher's language and the child's phonological processing. The analysis of these metrics may provide further insights into the neurobiological underpinnings of interaction.

The final article by Hojjati et al. aimed to predict the neuropsychological scores and investigate the non-linear progression trend of the cognitive declines based on multimodal neuroimaging data. The work utilized unimodal/bimodal neuroimaging measures to predict the neuropsychological scores in a large number of subjects (*n* = 1,143). This work shows an association between neuropsychological scores and sMRI and FDG-PET biomarkers from normal aging to severe AD.

Finally, five of the twenty-six submitted papers are accepted in this particular issue. The guest editors hope that the research contributions and conclusions in this Research Topic will help readers learn more about computational approaches used in neuroimaging and inspire them to work on various areas of these methods.

We sincerely thank the editor-in-chief for allowing us to organize this specific edition. We appreciate the help from the superb editorial office employees. We also acknowledge the reviewers' scholarly comments and all the authors who helped make this Research Topic possible.

## Author contributions

All authors listed have made a substantial, direct, and intellectual contribution to the work and approved it for publication.

